# A Peer Supervision Pilot Scheme

**DOI:** 10.1192/bjo.2022.140

**Published:** 2022-06-20

**Authors:** Laura Middleton, Jag Johal, Geraldine Swift

**Affiliations:** 1 Health Education England North West School of Psychiatry, Liverpool, United Kingdom; 2 Cheshire and Wirral Partnership NHS Foundation Trust, Chester, United Kingdom

## Abstract

**Aims:**

Following mixed feedback from Foundation (FY) trainees during their Psychiatry placements, Cheshire and Wirral Partnership NHS Trust introduced a pilot scheme whereby FY and General Practice (GP) trainees were paired with a current Psychiatry Core Trainee (CT). This was in addition to regular Clinical Supervisor meetings with weekly self-directed sessions encouraged. Suggestions included covering portfolio requirements or facilitating joint learning. The study aimed to improve FY/GP experience through increased learning and relationship-building opportunities. It equally offered leadership experience for CTs and development of supervision skills. Feedback gathered from the initial pilot would highlight difficulties and guide future peer supervision schemes.

**Methods:**

All FY/GP trainees allocated to the Trust from April–August 2021 were included. CTs were included by default but given the option to opt-out (opt-out policy). Pairings were made based on locality where possible (group sizes ranging from 2 to 3) and an initial supervisor training session was provided by the Director of Medical Education. Online feedback surveys were sent to all participants at baseline and after the pilot.

**Results:**

44 doctors were included in the pilot scheme, of which 26 completed the pre-pilot survey and 16 completed the post-pilot survey. Expected personal benefit prior to the scheme averaged 7.1/10, where 10 was deemed “extremely helpful” and 1 “not at all”. Following the scheme, experienced personal benefit was valued at 4.7/10. Reported benefits of the scheme were friendship, support and learning, although around a third described no benefit whatsoever. The most common problem encountered was that of being unable to meet and was seen in almost half of cases, with detailed feedback citing rota clashes or working on separate sites. Other problems included poor engagement and feeling the pairing was a poor match. Written feedback stated that the scheme was a good idea and well-supported, however there were challenges with its execution.

**Conclusion:**

Response rates were low throughout but particularly in the post-pilot survey, limiting interpretation. Overall, final scores did not appear to reflect initial optimism. The next cycle will include only CTs who have requested to be involved (opt-in policy), to establish whether this improves engagement. It will additionally incorporate a mid-point review to highlight and address issues at an earlier stage. The scheme is due to be repeated in 2022 and re-evaluated.

